# Regulation of Pre-rRNA Processing in Plant: Mechanisms, Plasticity, and Developmental Implications

**DOI:** 10.3390/plants15060940

**Published:** 2026-03-19

**Authors:** Nier Chen, Shiyi Huang, Beixin Mo, Wei Xiong

**Affiliations:** Guangdong Provincial Key Laboratory for Plant Epigenetics, Longhua Bioindustry and Innovation Research Institute, College of Life Sciences and Oceanography, Shenzhen University, Shenzhen 518060, China; nierchen1128@163.com (N.C.); shiyihuang11@163.com (S.H.)

**Keywords:** ribosomal DNA, pre-rRNA processing, plant development, quality control

## Abstract

Ribosome biogenesis is a fundamental process underlying plant growth, development, and environmental adaptation, and processing of precursor rRNA (pre-rRNA) represents one of its most critical regulatory steps. This review provides a systematic overview of the multi-layered regulatory mechanisms controlling pre-rRNA processing in plants, with *Arabidopsis thaliana* as the primary model system. We focus on the genomic organization of ribosomal DNA (*rDNA*) and its epigenetic regulation, illustrating how highly repetitive and sequence-diverse *rDNA* arrays maintain genomic stability while enabling tissue-specific expression of distinct *rDNA* variants. We further summarize the dynamic pathways of pre-rRNA processing and their plastic regulation under environmental conditions such as elevated temperature. In addition, we review the quality control systems that monitor pre-rRNA maturation, including non-templated tailing and exonuclease-dependent degradation pathways, which play essential roles in removing aberrant processing intermediates. We further examine how perturbations in pre-rRNA processing give rise to plant ribosomopathies and discuss complementary models of ribosome homeostasis and ribosome heterogeneity as frameworks for interpreting shared developmental phenotypes. Finally, by synthesizing genetic and molecular evidence, we highlight the pivotal role of pre-rRNA processing in orchestrating plant development and propose directions for future research.

## 1. Genomic Organization and Diversity of Genes Encoding rRNAs

*rDNA* is the genetic template for pre-rRNA transcription, and its genomic organization, sequence stability, and epigenetic regulation constitute the most upstream regulatory layer of plant ribosome biogenesis. In this section, we summarize the conserved structural characteristics of plant *rDNA* arrays, the molecular mechanisms maintaining their stability, the dosage buffering effect of redundant *rDNA* copies, and the epigenetic selection mechanism of *rDNA* sequence variants. These features of *rDNA* lay a genomic foundation for the subsequent regulation of pre-rRNA processing.

### 1.1. Organization, Stability, and Dosage Buffering of Plant rDNA Arrays

rRNA maturation must be tightly coordinated with the translational demands for plant growth and development, and both the transcriptional activity of *rDNA* and the regulation of pre-rRNA processing ultimately impact ribosome biogenesis. In plant genomes, hundreds of *rDNAs* are organized into multiple tandemly repeated gene clusters, known as nucleolus organizer regions (NORs), which are frequently positioned adjacent to heterochromatic domains [1,2,3]. Rather than random accumulations of repetitive sequences, on the contrary, plant NORs represent evolutionarily conserved, highly ordered, and functionally specialized chromatin units (reviewed in Sáez-Vásquez and Delseny, 2019) [4]. The intrinsic repetitiveness of *rDNA* units renders them particularly vulnerable to homologous recombination, replication stress, and transcription-replication conflicts, which can lead to *rDNA* copy number variation or structural rearrangements. To counteract these destabilizing forces, plant cells deploy tightly coordinated DNA repair and chromatin maintenance pathways to preserve NOR integrity [5,6,7]. For example, the RTR (RecQ-Top3-RMI) complex and the helicase RTEL1 act either independently or cooperatively to suppress deleterious homologous recombination events, thereby safeguarding the integrity of *rDNA* arrays (reviewed in Goffová and Fajkus, 2021) [8,9]. As a result, NORs exist in a state of dynamic equilibrium and their stability constitutes a prerequisite for appropriate *rDNA* transcription and proper nucleolar function. Notably, the organization of *rDNAs* is conserved from bryophytes to higher angiosperms [10,11,12]. In the early-diverging bryophyte *Physcomitrella patens*, the genome contains approximately 900 *rDNA* copies with a “linked” (L-type) arrangement in which the *5S* and 45S *rDNA* co-exist within the same tandem array. In contrast, most angiosperms exhibit a “separate” (S-type) organization with the *5S* and *45S rDNA* located in distinct clusters. For example, the monocot rice (*Oryza sativa*) carries ~850 copies of the *45S rDNA* repeat per diploid genome, forming a major nucleolar organizer region (NOR) at the telomeric end of the short arm of chromosome 9. In maize (*Zea mays*), the copy number of *rDNA* is even higher and more dynamic, ranging from ~5000 to 12,000 copies per haploid genome, mainly concentrated at the secondary constriction on the short arm of chromosome 6. Altogether, these observations indicate that the existence of large number of *rDNA* copies and the maintenance of a NOR chromatin state represent an evolutionary foundation for ribosome biogenesis and developmental regulation in plants [1,2,13].

A defining feature of plant NORs is their pronounced redundancy among *rDNA* copies. Remarkably, due to robust epigenetic compensation mechanisms of *rDNA* expression, CRISPR/Cas9-mediated deletion of approximately 90% of the *45S rDNA* copies in *Arabidopsis thaliana* neither compromises rRNA output nor causes growth and developmental abnormalities [14,15]. This dosage compensation is mediated by a reduction in repressive histone marks and large-scale reorganization of NOR architecture, indicating that cells can dynamically adjust the proportion of transcriptionally active *rDNA* units to meet the cellular demands for ribosome production [16,17]. This finding reveals one of the principal biological significances of existing expansive *rDNA* arrays: serving as a reservoir of backup copies while maintaining the nucleolar structure.

Such large-scale epigenetic coordination of *rDNA* activity occurs at the level of entire NORs and plays an important role in the establishment of nucleolar dominance [18], in which the complete NOR set inherited from one of the parents is coordinately silenced in interspecific or intraspecific hybrids [19,20,21]. Although the initiation of nucleolar dominance is stochastic, its outcome exhibits species bias and is shaped by complex epistatic interactions between parental *rDNA* haplotypes [21]. In polyploid wheat, the phenomenon of nucleolar dominance reaches an extreme form, whereby silenced parental *rDNA* arrays undergo progressive epigenetic modifications and are eventually eliminated from the genome, thereby promoting the stabilization of allopolyploid lineages [22]. This regulatory system enables plants to maintain homeostasis of ribosome biogenesis, while flexibly activating or repressing entire *rDNA* sets in response to developmental transitions, hybridization, or environmental stress [23].

### 1.2. rDNA Sequence Variation and Epigenetic Selection of rDNA Variants

Each *45S rDNA* repeat has its own promoter, and the precursors of 18S, 5.8S, and 25S rRNAs are co-transcribed from the *rDNA* loci forming a single long 45S rRNA. Sequences corresponding to mature rRNAs are separated by two internal transcribed spacers (ITS1 and ITS2) and flanked by external transcribed spacers (5′ ETS and 3′ ETS), whereas adjacent *45S rDNA* repeats are separated by intergenic spacers (IGSs) [4]. Although the sequences of mature rRNAs are highly similar in different *rDNA* units, they harbor intra-sequence polymorphisms, including single nucleotide polymorphisms (SNPs) and insertions/deletions (Indels). Specifically, SNPs in *25S rDNA* are predominantly located at NOR4, whereas SNPs/Indels in *18S rDNA* are mainly found at NOR2 [21,24]. Strikingly, the IGS and ETS regions exhibit pronounced sequence and structural variation. Length polymorphisms within these regions largely arise from variation in the *45S rDNA* copy internal subrepeats, driven by unequal homologous recombination events [25]. Such discrepancies are found not only between species but also among ecotypes, and even among individual *rDNA* units within a single genome, thereby constituting a rich reservoir of intergenomic and intragenomic polymorphisms [26,27,28]. These rapidly evolving spacer regions adopt specialized chromatin configurations [29,30]. For instance, the IGS region in *Arabidopsis* contains species-specific repetitive elements known as *Sal*I repeats [31]; while the IGS region in rice exhibits length polymorphism due to variable copies of ~254 bp and 62 bp subrepeats [11]. In addition, sequence variation within ITS1 is characterized by the presence or absence of an AvaI restriction site and a CAT trinucleotide insertion in ITS2, which together serve as molecular markers for distinguishing *Arabidopsis rDNA* variants. [21,24]. To date, most of these sequence polymorphisms and their chromatin-level implications have been characterized primarily in *Arabidopsis*. Functional characterization of *rDNA* arrays in other plant lineages remains largely unexplored.

Consistent with this diversity, multiple *45S rDNA* sequence variants, especially in the IGS and ETS regions, contribute to functional specialization and epigenetic regulation. Based on these variations, at least 74 distinct *rDNA* subtypes can be defined [24,32]. These variants are not randomly distributed within NORs, in contrast, the arrangement of them displays both chromosome specificity and spatial organization. For example, NOR2 on chromosome 2 and NOR4 on chromosome 4 harbor distinct subpopulations of *rDNA* variants [18]. Importantly, specific *rDNA* variants are preferentially transcribed in particular tissues, and the rRNAs they produce are successfully assembled into functional ribosomes [24]. The *rDNA* variant selection is governed by finely tuned epigenetic mechanisms. For example, the nucleolin-like protein AtNUC-L1 regulates symmetric DNA methylation at *rDNA* loci, thereby selectively activating or silencing distinct *rDNA* variants [33]. Similarly, the histone chaperone AtFKBP53 associates with chromatin to repress the expression of specific *rDNA* variants [34]. In addition, a complex composed of MBD and ACD proteins recognizes methylated *rDNA* and undergoes phase separation, subsequently recruiting the effector protein MORC6, thereby enabling selective regulation of different 45S rRNA gene variants [35]. Epigenetic status of *rDNA* variants directly determines their spatial partitioning: transcriptionally active, hypomethylated variants are enriched in the nucleolar interior, whereas inactive, hypermethylated variants are sequestered at the nucleolar periphery or in the nucleoplasm [9,36]. Such spatial partitioning ensures that required *rDNA* variants are deployed at the appropriate time and location.

**Figure 1:**
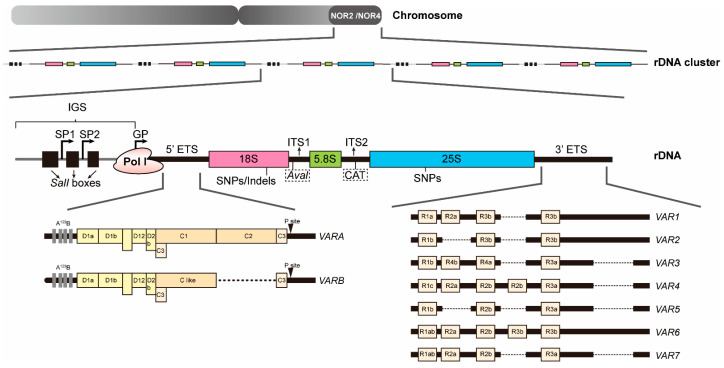
Genomic organization and structural diversity of plant rDNA. Schematic representation of the organization and sequence variation of *45S rDNA* units in *Arabidopsis*. Hundreds of rDNAs are organized into multiple tandemly repeated gene clusters on chromosome 2 and 4, respectively known as nucleolus organizer region 2 and 4 (NOR2 and NOR4), which are frequently positioned adjacent to heterochromatic domains. Each *rDNA* repeat contains the RNA polymerase I (Pol I) recognized promoter region, the 5′ external transcribed spacer (5′ ETS), the regions corresponding 18S, 5.8S and 25S mature rRNAs, two internal transcribed spacers (ITS1 and ITS2), and the 3′ external transcribed spacer (3′ ETS). Adjacent *45S rDNA* repeats are separated by intergenic spacers (IGSs). The IGS harbors multiple transcriptional regulating elements and repetitive sequences such as *Sal*I boxes. The IGS and ETS regions exhibit pronounced sequence and structural diversity. Based on these divergences, distinct *45S rDNA* variants (VARA, VARB, VAR1-VAR7) can be distinguished, which show non-random and chromosome-specific distributions among NORs. The *18S* and *25S rDNA* regions also harbor intra-sequence polymorphisms. In specific, the *18S* region contains single nucleotide polymorphisms (SNPs) and insertions/deletions (Indels), whereas the *25S* region contains SNPs. In the internal transcribed spacers, ITS1 variation is distinguished by the presence or absence of an AvaI restriction site, whereas ITS2 variation is marked by a specific trinucleotide insertion (CAT).

Collectively, plants have evolved a multilayered system to manage their *rDNA* repertoire by integrating genome architecture, copy number buffering, epigenetic regulation and *rDNA* variant selection. These *rDNA* variants are non-randomly distributed across NORs, exhibit chromosome-specific patterns, and are subject to tissue-specific epigenetic selection. Although solid experimental evidence is still needed to support the argument that *rDNA* variations may lead to functionally specialized ribosomes, it is proposed that pre-rRNA processing may represent a critical regulatory layer through which different *rDNA* variants might be selectively transcribed, and the resulting pre-rRNAs might be processed with different efficiency or differentially modified, thereby providing a potential mechanistic bridge between *rDNA* diversity and ribosome heterogeneity (Figure 1).

## 2. Dynamic and Alternative Pathways of Pre-rRNA Processing in Plants

Pre-rRNA processing is one of the core steps in ribosome biogenesis that links *rDNA* transcription to mature ribosome assembly. In this section, we systematically dissect both the canonical and the nonstandard processing routes of plant pre-rRNA, as well as the evolutionary conservation and lineage specificity of these pathways across different plant species. We also review evidence for dynamic switching between processing pathways in response to environmental stimuli, revealing the plasticity of plant pre-rRNA processing regulatory network.

### 2.1. Canonical and Alternative Pre-rRNA Processing Routes in Plants

Following RNA polymerase I (Pol I)-mediated transcription of *45S rDNA* within the nucleolus, the resulting 45S pre-rRNA undergoes a series of orchestrated processing events to generate the mature 18S, 5.8S, and 25S rRNAs [4,37].These processing events include a coordinated combination of endonucleolytic cleavages and exonucleolytic trimming executed by several ribonucleases, and assisted by RNA helicases, small nucleolar ribonucleoproteins (snoRNPs), and other auxiliary ribosome assembly factors [4,38,39]. Traditionally, pre-rRNA maturation has been depicted as a fixed pathway that is insusceptible to external and internal variables. However, increasing findings from plants challenge this view, revealing that pre-rRNA processing operates as a dynamic and regulatable network of alternative routes rather than a fixed sequence of events [38,40,41].

**Figure 2:**
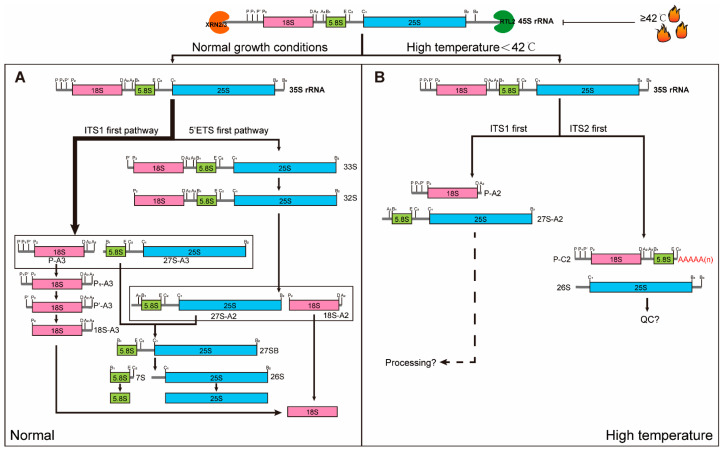
Temperature-dependent plasticity of pre-rRNA processing pathways. Alternative pre-rRNA processing pathways enable plants to adapt to changing environment in plants. The schematic illustrates the 45S pre-rRNA transcript and the cleavage sites targeted by the nuclease RTL2 and the 5′→3′ exoribonucleases XRN2/3. Following transcription in the nucleolus, the 45S pre-rRNAs are processed into the 35S pre-rRNAs via a series of endonucleolytic and exonucleolytic processing steps, mediated by nucleases such as RTL2 and the 5′→3′ exoribonucleases XRN2/3. (**A**) Under normal growth condition, most mature rRNAs are generated from the canonical ITS1-first and 5′ ETS-first pathways, giving rise to intermediates such as P-A3, 18S-A2 and 27S-A2/A3. (**B**) Under moderate heat stress (below 42 °C), the 35S pre-rRNA processing pathways are remodeled: the ITS2-first pathway, characterized by the accumulation of specific intermediates such as P-C2 and 26S pre-RNAs, runs in parallel with the ITS1-first pathway, which produces P-A2 instead of P-A3. The shift toward the ITS2-first pathway probably triggers rRNA quality control mechanisms or slow the processing rate in response to heat stress. Upon return to normal conditions, the canonical processing pathways are restored. When the temperature exceeds 42 °C, the processing efficiency of the 45S pre-rRNA is substantially reduced compared with that under normal condition.

During transcription, RTL2 mediates a co-transcriptional cleavage at the B0 site within the 3′ ETS of the 45S pre-rRNA, thereby defining the 3′ boundary of the transcript [42,43]. The resulting precursor is then subjected to 5′-end trimming by the 5′ → 3′ exoribonucleases XRN2 or XRN3 [37,44]. Following this exonucleolytic processing, a precise endonucleolytic cleavage directed by U3 snoRNA in cooperation with NUC1 gives rise to the 35S pre-rRNA [4,37]. Under normal growth conditions, most plant cells predominantly employ 35S pre-rRNA processing pathways that largely resemble those described in animals (the ITS1-first pathway) and yeast (the 5′ ETS–first pathway) [4,37,45]. In the ITS1-first pathway, cleavage within ITS1 (site A3) directly separates the precursors for the small and large ribosomal subunits. In contrast, the 5′ ETS-first pathway initially removes the 5′ ETS to generate the 32S intermediate, which subsequently undergoes cleavage in ITS1 (site A2) to separate the subunits. Both pathways eventually lead to the stepwise removal of ITS2 to generate mature 18S, 5.8S, and 25S rRNAs [4]. These pathways ensure efficient production of the small and large ribosomal subunits to support rapid cell proliferation during vegetative growth (Figure 2A). In addition to these two canonical routes, a plant specific pre-rRNA processing pathway termed the ITS2-first pathway has been reported. In this pathway, a primary cleavage occurs at the C2 site within ITS2, generating intermediates such as P-C2, which encompasses both 18S and 5.8S rRNAs [37]. This ITS2-first pathway has been documented in several plant species, including *Arabidopsis*, rice and *Solanum lycopersicum* [41]. It appears to operate preferentially in rapidly proliferating cells and can be activated under stress conditions such as elevated temperature [38,40].

### 2.2. Environmental Modulation and Reversible Switching of Processing Pathways

As mentioned above, environmental stimuli such as high temperature could induce the non-standard ITS2 first pre-rRNA processing pathway, which manifests that this critical physiological process is under dynamic modulation [40,41]. Time-resolved Northern blot analyses of heat-treated seedlings reveal a transient and pronounced accumulation of intermediates associated with the ITS2-first pathway, concomitant with a reduction in polysome abundance. Notably, this stress-induced processing state is reversible. Upon return to permissive temperatures, canonical processing intermediates reappear within hours, and normal ribosome assembly is restored. This reversibility strongly supports the existence of an actively regulated switching mechanism.

In addition, dynamic remodeling of processing pathways has also been observed under cold stress [46] and conditions of sugar imbalance [47], often correlating with changes in the expression or activity of specific processing factors and RNA helicases [48]. Under cold stress, pre-rRNA processing is globally delayed in both rice and *Arabidopsis*, leading to the accumulation of unprocessed 45S transcripts and reduced canonical pathway intermediates. It should be noted that, unlike heat stress, cold stress does not activate the ITS2 first pre-rRNA processing pathway [41,46]. Under conditions of sugar imbalance, glycolysis-linked cellular energy status regulates ribosome biogenesis via the TOR pathway [49,50].

Collectively, these observations establish pre-rRNA processing as a responsive regulatory interface through which environmental signals can rapidly modulate translational capacity. However, the exact mechanism of alternative pathway switch under heat stress remains unclear, and whether more stresses could lead to this phenomenon needs to be investigated.

## 3. Pre-rRNA Processing Quality Control

Aberrant and over-accumulated middle products during pre-rRNA maturation need to be eliminated, and this physiological process is executed via rRNA quality control pathways. This section will elaborate on how abnormal processing intermediates are targeted by the rRNA surveillance pathways.

### 3.1. Generation and Recognition of Aberrant Pre-rRNA Intermediates

During rRNA maturation, defective processing intermediates might be generated, as it is a highly complicated biological process involving numerous factors [51]. In addition, abnormal processing intermediates can accumulate excessively in mutants of factors related to pre-rRNA processing or in situations where processing efficiency is reduced under stress conditions [52]. Therefore, cells have evolved an efficient quality surveillance system to eliminate improperly processed and over-accumulated pre-rRNAs, ensuring the accurate production of mature rRNAs.

A main feature of many pre-rRNA processing intermediates in plants is non-templated 3′ tailing. These non-templated 3′ tailings can be polyadenylation, polyuridylation, or mixed nucleotides, depending on types of intermediates and genetic background [53,54]. Adenylation is the most frequently observed 3′ modification on intermediates such as excised 5′ ETS fragments and stalled 18S-A3 precursors [41,54]. Uridylated 18S-A2 intermediates are markedly increased in the *rrp6l2* mutant [54], and tails containing cytidines and guanosines have also been detected on a small subset of precursors [53]. Different types of non-templated 3′ tailing indicate that “tailing modifications” maybe result from multiple tailing enzymes. However, it remains unclear whether these tail modifications function solely as signals for rRNA quality control or whether they also act as specialized markers during rRNA processing.

In yeast (*Saccharomyces cerevisiae*), pre-rRNA tailing is mainly mediated by the TRAMP complex (Trf4/5-Air1/2-Mtr4), which marks defective RNAs for degradation [55,56]. In plants, non-templated tailings on some rRNA intermediates are catalyzed by the TRL terminal nucleotidyl transferase, which is a functional homolog of yeast Trf4/Trf5, and primarily catalyzes adenylation of the 5′ ETS fragments, 5.8S-C2, and 18S-A3 intermediates, thereby promoting their recognition by the RNA surveillance machinery [54]. The observed diversity of 3′ tailing patterns suggests that additional terminal nucleotidyl transferases may act on rRNA termini in *Arabidopsis*. This point of view is further supported by the observation that uridylation of the 18S-A2 fragment persists or is even enhanced in the *trl* mutants [53,54]. Among the currently characterized several terminal nucleotidyl transferases, only two, HESO1 and URT1, have been shown to have uridyltransferase activity. However, whether these two uridyltransferases participate in tailing precursor rRNA intermediates remains unknown [57,58,59].

### 3.2. Surveillance Pathways and Functional Consequences of Pre-rRNA Quality Control

The removal of these tailed substrates is coordinated by a set of exonucleases. The nuclear-localized exosome complex, assisted by the RNA helicase MTR4, generally targets adenylated substrates for 3′→5′ degradation [60,61]. Meanwhile, the exonuclease RRP6L2 appears to preferentially decay uridylated intermediates, such as 18S-A2 [54,62]. It is believed that additional exonucleases may be responsible for degradation of substrates of rRNA intermediate with other tailing patterns. However, existing studies have not provided detailed analyses of pre-rRNA 5′ends during degradation, and thus it remains unproven whether bona fide 5′→3′ rRNA degradation occurs [37]. In yeast, pathway of rRNA degradation is a “turnover cycle” rather than a simple linear process. Degradation intermediates derived from pre-rRNAs often carry longer poly(A) tails, indicating that these RNAs resist initial exosome-mediated degradation and subsequently undergo additional rounds of extensive “re-polyadenylation” before being re-engaged by the exosome for further degradation [62]. This cyclical licensing mechanism ensures persistent marking and repeated targeting of rRNA substrates until their complete elimination, thereby preventing the accumulation of potentially toxic RNA fragments. Although plants have homologs of key players involved in yeast nuclear surveillance system, it remains to be validated that whether the “turnover cycle” pathway of rRNA degradation is applied in plants [54,55,60].

**Figure 3:**
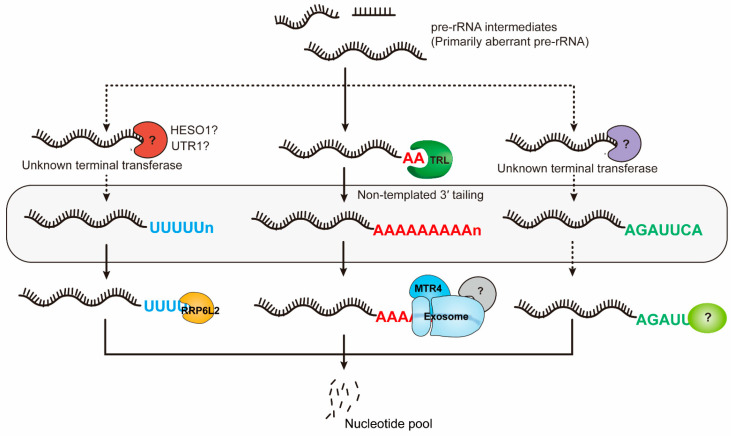
Quality control and degradation pathways of pre-rRNA processing intermediates in plants. During rRNA maturation, erroneously processed or excessively accumulated pre-rRNA intermediates are recognized and eliminated by the nuclear RNA surveillance machinery. A prominent feature of pre-rRNA intermediates is non-templated 3′ tailing, which includes polyadenylation, polyuridylation, and mixed types. Different types of 3′ tailing are likely catalyzed by different terminal nucleotidyl transferases. Specifically, TRL predominantly mediates adenylation of specific pre-rRNA intermes such as excised 5′ ETS fragments and 18S-A3, whereas enzymes responsible for uridylation or mixed tailing are still uncertain (indicated by question marks). Tailed pre-rRNA intermediates are selectively eliminated through distinct decay pathways. Adenylated substrates are generally targeted for 3′→5′ degradation by the nuclear exosome, assisted by the RNA helicase MTR4, whereas uridylated intermediates, such as 18S-A2, are preferentially degraded by the exonuclease RRP6L2. The nucleases responsible for eliminating pre-rRNA intermediates with other tailing patterns remain to be identified. Through this coupling of tailing-based marking with tail-specific degradation pathways, plant cells efficiently prevent the accumulation of aberrant pre-rRNAs and maintain rRNA homeostasis during ribosome biogenesis.

Together, these observations indicate that rRNA quality control in plants functions as a selective filtering system rather than merely a waste disposal mechanism. By coupling processing intermediates to diverse tail modification signals and deploying exonucleases in a tail-specific manner, plant cells may actively shape the population of pre-rRNAs to maintain necessary ribosome assembly. As mentioned earlier, specific rDNA variants are preferentially transcribed in specific tissues [24]. An outstanding question is whether the rRNA quality control machinery contributes to this selectivity by eliminating rRNA variants that are inappropriate for expression in certain tissues. Besides disposing of defective rRNAs, the rRNA tailing system also serve as a screening mechanism for tissue-specific expression of rRNA variants. Ribosome biogenesis is an inherently complex and error-prone biological process, yet our understanding of the rRNA surveillance mechanisms governing ribosome assembly remains limited (Figure 3).

## 4. Concerted Control of Pre-rRNA Maturation by a Diverse Factor Network

Pre-rRNAs, transcribed from the repetitive *rDNA* arrays, undergoes a tightly coordinated maturation process that requires the collaborative action of multiple types of regulatory factors including epigenetic regulators, ribonucleoproteins, nucleases, RNA helicases, and ribosomal proteins (RPs). These factors form a complex and interconnected molecular network to ensure the accuracy and efficiency of pre-rRNA processing. This section focuses on the upstream regulatory determinants of *rDNA* transcription and the mechanistic network governing pre-rRNA processing.

### 4.1. rDNA Transcriptional State as an Upstream Determinant of Pre-rRNA Processing

The efficiency of *rDNA* transcription determines the abundance of 45S pre-rRNAs and thus influence subsequent processing steps. Thus, *rDNA* epigenetic status, chromatin architecture, and intrinsic DNA secondary structures collectively establish an upstream regulatory framework which links rDNA transcription to rRNA maturation.

Among the epigenetic features of *rDNA*, DNA methylation constitutes a central regulatory layer in *Arabidopsis*, with CG and CHG methylation maintained by MET1 and CMT3, respectively [63]. Methylated *rDNA* is recognized by the MBD-ACD complex, in which MBD5 and MBD6 directly bind CG-methylated DNA, while the α-crystallin domain proteins ACD15.5 and ACD21.4 promote liquid–liquid phase separation to stabilize complex enrichment at NORs [35]. Subsequent recruitment of the ATPase MORC6 to methylated *rDNA* locus induces chromatin compaction through ATP-dependent remodeling, the consequence of which is restriction of Pol I accessibility and transcriptional silencing [35,64]. Recognition of CHG methylation is primarily mediated by SRA-domain-containing proteins, including members of the SUVH family of histone methyltransferases, which bind methylated CHG sites and maintain H3K9me2, thereby establishing a reinforcing feedback loop between DNA methylation and histone modification [65].

Histone modifications further constrain *rDNA* transcription by controlling chromatin accessibility and Pol I processivity. HDA6 represses *rDNA* transcription by removing activating acetyl marks, limiting Pol I recruitment and elongation while preventing aberrant RNA Pol II transcription within intergenic spacer regions [66,67]. HD2B, a plant-specific histone deacetylase, is recruited to *rDNA* promoter through direct interaction with RPS6, where it removes histone acetylation, resulting in transcriptional repression [50,68]. Histone methylation factors involved in *rDNA* regulation mainly include SUVH5/6, ATXR5/6, and SUVR4. SUVH5/6 and ATXR5/6 deposit H3K9me2 and H3K27me1, respectively, reinforcing *rDNA* silencing [17,65,69], whereas SUVR4 contributes to *rDNA* repression primarily in the context of nucleolar dominance in hybrids [70].

The three-dimensional organization of *rDNA* is dynamically regulated by antagonistic chromatin-associated factors that influence *rDNA* transcription. The nucleolar protein NUC1 binds *rDNA* promoters and maintains an active chromatin configuration, characterized by reduced DNA methylation and diminished heterochromatic marks, thereby facilitating Pol I engagement [33,71]. Loss-function of NUC1 shifts *rDNA* toward a heterochromatic state. The function of NUC1 is counteracted by NUC2, which promotes transcriptional repression of *rDNA* [72]. The chromatin remodeler DDM1 enables DNA methyltransferase access to compacted chromatin, the consequence of which is reinforcement of *rDNA* condensation [73]. The AAA-ATPase CDC48A mediates developmental stage-specific disassembly of centromeric heterochromatin, promotes release of *rDNA* arrays into the nucleolus during pollen development thus licenses their transcription [74].

**Figure 4:**
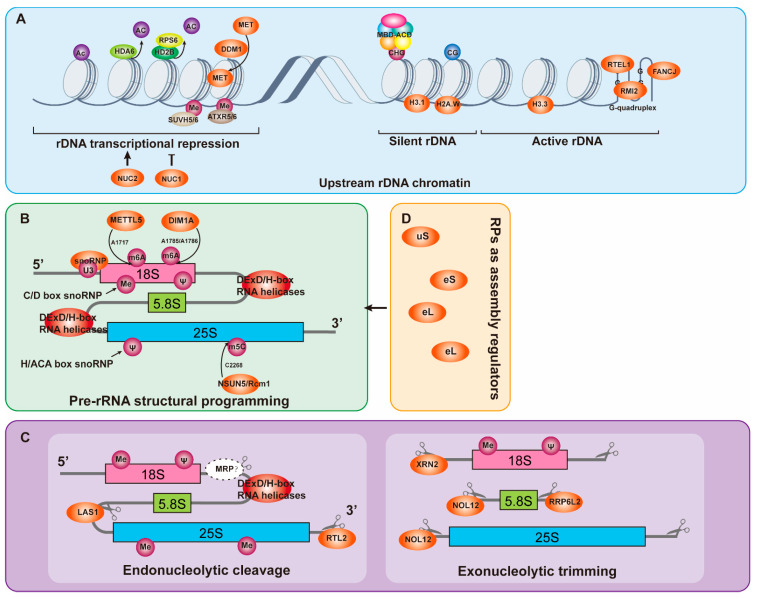
Network control of pre-rRNA maturation in plants. (**A**) The epigenetic and structural determinants of *rDNA* transcription. The left side depicts transcriptional repression complexes including histone deacetylases (HDA6, HD2B), histone methyltransferases (SUVH5/6, ATXR5/6), and chromatin remodeling factors (DDM1, NUC1/2), which collectively promote heterochromatin formation. The right side shows the silent and active states of *rDNA*. Silent *rDNA* is enriched in heterochromatin-associated histone variants such as H3.1 and H2A.W and exhibits high levels of DNA methylation. In contrast, transcriptionally active *rDNA* regions contain the histone variant H3.3 and may form potential G-quadruplex structures whose stability is regulated by helicases including RTEL1, RMI2, and FANCJ. (**B**) Pre-rRNA structural folding and chemical modification. Newly transcribed pre-rRNAs undergo extensive folding and chemical modifications. Key modifications include m^6^A methylation catalyzed by METTL5 and DIM1A; pseudouridylation (Ψ) and 2′-O-methylation guided by small nucleolar ribonucleoproteins (snoRNPs); m^5^C methylation mediated by NSUN5/Rcm1. In addition, DExD/H-box RNA helicases facilitate proper pre-RNA folding, facilitating subsequent processing events. (**C**) Endonucleolytic cleavage and exonucleolytic trimming of pre-RNAs. The left portion illustrates endonucleolytic cleavage events, mediated by nucleases such as LAS1 and RTL2, at defined processing sites including the ITS2 and 3′ ETS regions. The right portion shows exonucleolytic trimming of pre-RNAs by exonucleases including XRN2 (5′ → 3′ direction), NOL12, and the RNA exosome component RRP6L2 (3′ → 5′ direction), thereby removing spacer sequences and generating the mature 18S, 5.8S, and 25S rRNA termini. (**D**) Ribosomal proteins (RPs) in pre-rRNA maturation. RPs function not only as structural components of the ribosome but also as regulatory factors during pre-rRNA maturation. Several RPs associate with nascent rRNA to stabilize RNA structures and facilitate early-stage processing ensuring the efficient and accurate assembly of ribosomal subunits.

Histone modification patterns also modulate *rDNA* chromatin configuration by influencing nucleosome stability and higher-order folding. H3.1 and H2A.W associate with heterochromatic chromocenters and H3K9me2 to compact silent *rDNA* arrays and restrict their transcription [9,75]. By contrast, H3.3 is enriched at active *rDNA* promoters to facilitate Pol I elongation [75]. Transcriptionally active *rDNA* arrays assemble into compact nucleolar foci enriched in H3.3 and depleted of H3K9me2, indicating that histone modification patterns regulate both transcriptional output and physical folding of *rDNA* arrays [9]. Even in the open chromatin, *rDNA* transcription can be impeded by specific DNA secondary structures. For instance, the GC-rich *rDNA* sequence favors G-quadruplex (G4) formation, which obstructs Pol I progression. Helicases such as RTEL1, RMI2, and FANCJ resolve these special configuration of *rDNA*, and inactivation of one of these helicases will cause replication stress, *rDNA* damage, and *rDNA* copy number instability, which leads to disorder of *rDNA* transcription [1,7,8] (Figure 4A).

### 4.2. Mechanistic Network Governing Pre-rRNA Processing in Plants

Newly transcribed 45S rRNAs rapidly fold with complex higher-order structure, and subsequently undergo site-specific chemical modifications that function as structural anchors. In plants, these modifications are mediated primarily by two classes of small nucleolar ribonucleoprotein (snoRNP) complexes: C/D box snoRNPs, centered on fibrillarin (FIB1/2) and catalyzing 2′-O-ribose methylation; and H/ACA box snoRNPs, centered on NAP57 and responsible for pseudouridylation [76,77,78]. In parallel, several stand-alone enzymes also mediate specific modifications at key functional positions of rRNAs. For instance, DIM1A catalyzes m^6^_2_A modification of A1785/A1786 in 18S rRNA [79]; NSUN5/Rcm1 mediates m^5^C modification of C2268 in 25S rRNA [4,80] (review in Julio Sáez-Vásquez and Michel Delseny, 2019); and METTL5 catalyzes m^6^A modification of A1717 in *Arabidopsis* 18S rRNA [81]. U3 snoRNP, one of the C/D box snoRNPs, represents a specialized module that dictate pre-rRNA folding and processing. Within U3 snoRNP, the U3 small nucleolar RNA associates with specific proteins such as RRP9, MPP10, IMP3, and IMP4, and its 5′ region base-pairs with the 5′ external transcribed spacer (5′ ETS) of pre-rRNA, thus stabilizing defined folding intermediates and positioning cleavage sites [4,82,83,84]. As a core component of the small subunit (SSU) processome, or 90S pre-ribosome, U3 snoRNA guides cleavage site selection while acting as an RNA chaperone to prevent misfolding during early assembly [82,85,86] (Figure 4B).

In addition to modifications, the major events of pre-rRNA maturation are endonucleolytic cleavage and exonucleolytic trimming. Endonucleases RTL2, NOB1, and LAS1 catalyze cleavage at the 3′ ETS, the D site defining the 3′ end of 18S rRNA, and the C2 site within ITS2, respectively [54,87,88]. Endonuclease MRP may perform ITS1 cleavage in plant, although further evidence is required [54,89]. Among exonucleases, the 5′-3′ exonuclease XRN2 trims the 5′ ETS to expose the P site which is required for U3 snoRNP dependent cleavage, while the 5′-3′ exonuclease NOL12 participates in 27S precursor processing and 5.8S rRNA 5′ end maturation [37,40,90]. The 3′-5′ exonucleolytic trimmings are executed by the RNA exosome with assistance of RRP6L2, ensuring accurate 3′ end formation and removal of processing by-products. In plants, execution of these 3′ → 5′ exonucleolytic trimming steps by the exosome not only relies on the hydrolytic nuclease RRP6L2, but also involves a unique phosphorolytic activity of its core subunit RRP41, which directly contributes to the degradation and processing of specific substrates, such as 5.8S rRNA and 5′ ETS-derived by-products [4,40,91] (Figure 4C).

Different types of DExD/H-box RNA helicases resolve inhibitory RNA structures to facilitate endonucleolytic cleavage and exonucleolytic trimming. Specifically, several DEAD-box helicases function as non-processive RNA chaperones [92,93], whereas a few DExH-box helicases unwind highly structured regions to enable nuclease access and snoRNA release [55,60]. rRNA helicases function throughout the entire process of ribosome biogenesis and can be broadly classified into five major roles: regulation of snoRNA association and release, facilitation of pre-rRNA cleavage, assistance in exosome-mediated RNA degradation and trimming, RNA chaperone activity, and integration of ribosome biogenesis with plant developmental and signaling pathways.

During ribosome biogenesis, RPs serve not only as the structural skeleton of the ribosome but also act as critical trans-acting factors and RNA chaperones to regulate the folding and processing of pre-rRNA [79,94]. RPs typically associate with nascent rRNA in a 5′ to 3′ order, early-binding RPs stabilize rRNA structures to expose cleavage sites, while late-binding RPs (such as RPs located near the polypeptide exit tunnel of the large subunit) are responsible for recruiting specific non-ribosomal assembly factors [95]. Furthermore, like rDNAs, the transcription and modification of RPs are also dynamically regulated in response to environmental changes, once again indicating that ribosome biogensis is a dynamic process [95,96] (Figure 4D).

## 5. Impact of rRNA Processing on Plant Development

The generation of mature rRNAs is an evolutionarily conserved core process for ribosome biogenesis, and its functional integrity is a prerequisite for plant growth and development across diverse plant taxa. The impairment of pre-rRNA processing pathways could lead to severe developmental consequences.

### 5.1. Developmental Consequences of Disrupted rRNA Processing

rRNA maturation is pivotal to ribosome biogenesis which constitutes a core cellular process underlying plant growth and development. Disruption of factors involved in ribosome biogenesis frequently results in embryonic lethality, whereas hypomorphic mutants of these factors exhibit similar pleiotropic developmental defects with subtle variations. Collectively, these phenotypes are referred to as plant ribosomopathies [97].

The phenotypic manifestations of plant ribosomopathies are predominantly associated with abnormalities in leaf morphology and floral organ development. Leaf defects typically include narrowed blades, pointed leaf tips, and disrupted vascular patterning, and in some mutants, serrated leaf margins are also observed. Notably, these leaf phenotypes are highly conserved across multiple plant species including *Arabidopsis*, rice, maize and wheat [98,99]. Floral defects are commonly characterized by irregular numbers and sizes of sepals and are frequently accompanied by compromised reproductive capacity or sterility. Defects in pre-rRNA processing also frequently impair root development, resulting in shortened primary roots, overall reduced root length, and defects in lateral root initiation or elongation. Such phenotypes have been reported in multiple ribosome-related mutants, including SAHY1 [100], HD2B/HD2C [68], APUM23 [101] and DXO1 [102], all of which display reduced primary root growth. In contrast, loss of RFC3 [103] or UTP18 [104] predominantly affects lateral root formation or elongation. Beyond organ growth, several pre-rRNA processing factors are directly implicated in cell fate determination. For instance, mutation of DIM1A disrupts the differentiation pattern between trichoblast and atrichoblast cells in the root epidermis [79], whereas loss of UTP18 or PCN results in polyembryony [104].

### 5.2. Ribosome Homeostasis, Heterogeneity, and Environmental Modulation of Development

An important question arising from these observations is why different mutants affecting ribosome biogenesis show similar stereotypical developmental abnormalities. Two major hypotheses have been proposed to explain this phenomenon. (1) The ribosome homeostasis hypothesis posits that plant development is highly sensitive to the total abundance of functional ribosomes. When the rate of ribosome biogenesis is slowed down and total amount of functional ribosomes is reduced, translation of different mRNAs might not be equally affected, translation of some specific mRNAs might be particularly sensitive to reduced ribosome availability, thereby reshaping developmental programs [105,106]; (2) An alternative, though not mutually exclusive, explanation is the ribosome heterogeneity hypothesis, which proposes the existence of “specialized ribosomes”. Heterogenous ribosomes, arising from divergent ribosomal components, may preferentially translate specific subsets of mRNAs or modulate development through translation-dependent or translation-independent signaling pathways [107]. Martínez et al. have argued that the ribosome homeostasis model should be regarded as a parsimonious null hypothesis, while acknowledging that both mechanisms may coexist and operate in different biological contexts [97].

Since plant genomes harbor hundreds of redundant *rDNA* copies and *rDNA* dosage variation can be buffered through epigenetic regulation, the rate of ribosome biogenesis is not limited by *rDNA* dosage but is instead constrained by the efficiency of *rDNA* transcription and pre-rRNA processing [23,71,108]. Pre-rRNA maturation requires a series of tightly coordinated processing steps, and disruption of any of these steps will reduce ribosome biogenesis efficiency. When the total amount of functional ribosomes falls below the threshold required to sustain rapid cell proliferation, ribosomopathy phenotypes ultimately arise. Ribosomopathy-associated defects are most pronounced in tissues with high proliferative and differentiation activity, such as leaf primordia, floral primordia, and the root apical meristem show the most pronounced ribosomopathy phenotypes. These tissues are demonstrated to have relative higher protein synthesis demand and mRNAs encoding key developmental regulators are actively translated. Interestingly, mRNAs of a subset of these regulators contain one or more upstream open reading frames (uORFs) in their 5′ untranslated regions (5′-UTRs) [109,110].When functional ribosomes are abundant, translational re-initiation at the downstream main open reading frame (mORF) occurs more frequently than under conditions of limited ribosome availability [97].Therefore, uORF-containing mRNAs are much more sensitive to defects in ribosome biogenesis caused by impaired pre-rRNA processing, which leads to specific developmental abnormalities. In this context, ribosome heterogeneity offers an important complementary explanation.

**Figure 5:**
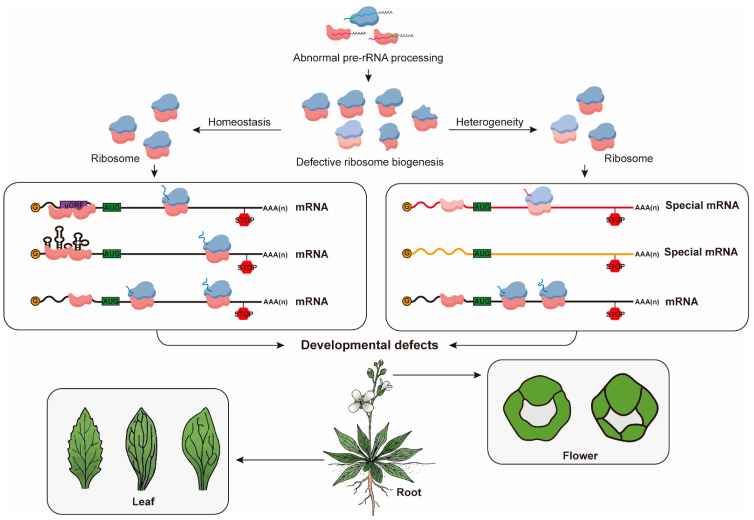
Developmental abnormalities resulted from impaired pre-rRNA processing pathways. Malfunction of pre-rRNA processing disturbs ribosome homeostasis, leading to two non-mutually exclusive scenarios: a reduction in total functional ribosome abundance (ribosome homeostasis model) or altered composition of ribosome populations due to impaired biogenesis of specific ribosome variants (ribosome heterogeneity model). In the homeostasis model, a global reduction in functional ribosomes disproportionately impairs the translation of ribosome-sensitive mRNAs, particularly those containing upstream open reading frames (uORFs) in their 5′-UTRs, which require efficient ribosome re-initiation, while standard mRNA translation remains relatively unaffected. In the heterogeneity model, the defect leads to a depletion of specialized ribosome subpopulations. Consequently, the translation of several special mRNAs that are strictly depend on these specific ribosome variants for their efficient initiation is compromised, whereas other mRNAs are translated normally by the remaining ribosome pool. These distinct translational dysregulations ultimately converge to cause similar developmental defects, including leaf morphological alterations (e.g., narrowed blades, serrated margins), root growth defects (e.g., shortened primary roots), and floral structural anomalies (e.g., irregular sepal number, sterility).

The existence of extensive *rDNA* sequence variation and diverse epitranscriptomic modifications of rRNA provides compelling evidence for ribosome heterogeneity in plants [95]. For example, Simona Krassnig et al. demonstrated that distinct *rDNA* variants are differentially transcribed and assembled into polysomes in a tissue-specific manner, with certain variants predominating in roots and others in floral buds, providing strong evidence for the presence of tissue-specific ribosomes in plants [24]. Moreover, METTL5-mediated m^6^A modification of 18S rRNA alters ribosome structure and enhances translation of ABA-responsive genes [81,111]. Besides the heterogeneity caused by *rDNA* variants, the exitance of paralogous RP genes also contribute componential diversity to ribosomes, which may further enhance adaptation to complex developmental processes and environmental stresses through selective translation in plants. Taken together, these findings suggest that ribosome homeostasis and ribosome heterogeneity likely coexist within plant cells. Pre-rRNA processing efficiency establishes ribosome homeostasis and defines the upper limit of translational capacity during development, while selective transcription of *rDNA* variants and their differential fates during processing and quality control enable the generation of ribosome populations with potential functional specialization, without globally perturbing translational balance (Figure 5).

## 6. Conclusions and Perspectives

Over the past decade, studies of plant ribosome biogenesis have substantially revised the traditional view of this process as a constitutive housekeeping activity. It is now clear that ribosome biogenesis is highly regulated and tightly integrated with plant development and environmental responsiveness. Plant cells operate a multi-layered regulatory framework in which large and heterogeneous *rDNA* arrays are epigenetically buffered, specific *rDNA* variants are differentially transcribed across tissues, pre-rRNAs are processed through context-dependent pathways, and maturation intermediates are subjected to stringent quality-control mechanisms. Together, these layers ensure robust ribosome production while permitting flexibility in translational output.

Several important questions remain open. First, although tissue-specific expression of *rDNA* variants has been documented, direct evidence that such variants give rise to ribosomes with distinct translational preferences in plants is still limited. Addressing this question will require experimental strategies that combine high-resolution ribosome profiling with targeted genetic manipulation of defined *rDNA* variants, enabling rigorous tests of the specialized ribosome hypothesis. Second, the molecular mechanisms that govern the switch between canonical and alternative pre-rRNA processing pathways, particularly under stress conditions, remain poorly understood. Identifying the upstream signals, sensors, and regulatory components that impinge on the processing machinery will be essential for understanding how environmental cues are translated into quantitative changes in ribosome output. Third, the relationship between rRNA surveillance pathways and *rDNA* variant usage represents an emerging area of interest. It is conceivable that quality-control systems do more than eliminate aberrant pre-rRNAs, and instead contribute to the selective retention or exclusion of specific rRNA variants in a context-dependent manner. Testing this possibility will require combined analysis of surveillance components and rRNA variant fate and incorporation. Finally, linking defects in ribosome biogenesis to discrete developmental outcomes remain a major challenge. Progress in this area will likely depend on integrative, systems-level approaches that combine transcriptomic, translatomic, and metabolic analyses in ribosomopathy mutants. Such studies should help identify subsets of mRNAs whose translation is most sensitive to ribosome abundance or composition, thereby providing a direct mechanistic connection between nucleolar function and plant morphogenesis.

In conclusion, the field is transitioning from descriptive characterization toward mechanistic understanding. Elucidating how the progression from *rDNA* transcription to functional ribosome assembly is regulated will be central to explaining how ribosome biogenesis contributes to developmental robustness and environmental adaptation in plants.

## Data Availability

No new data were created or analyzed in this study.
